# Molecular Mechanisms Underlying Curcumin-Mediated Therapeutic Effects in Type 2 Diabetes and Cancer

**DOI:** 10.1155/2018/9698258

**Published:** 2018-03-20

**Authors:** Marzena Wojcik, Michal Krawczyk, Pawel Wojcik, Katarzyna Cypryk, Lucyna Alicja Wozniak

**Affiliations:** ^1^Department of Structural Biology, Faculty of Biomedical Sciences and Postgraduate Education, Medical University of Lodz, ul. Zeligowskiego 7/9, 90-752 Lodz, Poland; ^2^Department of Horticultural Crop Management and Nutrition, Research Institute of Horticulture, ul. Konstytucji 3 Maja 1/3, 96-100 Skierniewice, Poland; ^3^Diabetology and Metabolic Diseases Department, Medical University of Lodz, ul. Pomorska 251, 92-213 Lodz, Poland

## Abstract

The growing prevalence of age-related diseases, especially type 2 diabetes mellitus (T2DM) and cancer, has become global health and economic problems. Due to multifactorial nature of both diseases, their pathophysiology is not completely understood so far. Compelling evidence indicates that increased oxidative stress, resulting from an imbalance between production of reactive oxygen species (ROS) and their clearance by antioxidant defense mechanisms, as well as the proinflammatory state contributes to the development and progression of the diseases. Curcumin (CUR; diferuloylmethane), a well-known polyphenol derived from the rhizomes of turmeric *Curcuma longa*, has attracted a great deal of attention as a natural compound with beneficial antidiabetic and anticancer properties, partly due to its antioxidative and anti-inflammatory actions. Although this polyphenolic compound is increasingly being recognized for its growing number of protective health effects, the precise molecular mechanisms through which it reduces diabetes- and cancer-related pathological events have not been fully unraveled. Hence, CUR is the subject of intensive research in the fields Diabetology and Oncology as a potential candidate in the treatment of both T2DM and cancer, particularly since current therapeutic options for their treatment are not satisfactory in clinics. In this review, we summarize the recent progress made on the molecular targets and pathways involved in antidiabetic and anticancer activities of CUR that are responsible for its beneficial health effects.

## 1. Introduction

Type 2 diabetes mellitus (T2DM) and cancer are growing at epidemic proportions resulting in significant economic, social, and health burden. Moreover, there is increasing incidence of obesity that is associated with an increased risk of developing T2DM and cancer [1]. Despite many efforts, the current therapeutic options for the treatment of T2DM and cancer remain far from satisfactory, primarily because of occurring serious side effects. Therefore, new therapeutic strategies are continually being searched to better treat the diseases. In the last years, a considerable attention has been focused on investigating functional properties of plant-derived compounds for their potential therapeutic purposes.

Curcumin (CUR) [1,7-bis(4-hydroxy-3-methoxyphenyl)-1,6-heptadiene-3,5-dione] also called diferuloylmethane is a polyphenolic compound derived from the rhizomes of turmeric *Curcuma longa* that is well known not only as a spice in Asian cuisine and a food preservative agent but also as a phytochemical compound with the beneficial effects on several chronic disease states associated with inflammation and oxidative stress, including T2DM and cancer.

Oxidative stress is an imbalance between the formation of reactive oxygen species (ROS) and the antioxidant defense mechanisms in the cell which results in ROS-mediated peroxidation of membrane lipids and oxidative damage of DNA and proteins. There is now overwhelming evidence that CUR at low concentrations is a strong antioxidant that scavenges ROS, decreases lipid peroxidation, and stimulates antioxidant enzymes, including catalase, superoxide dismutase (SOD), glutathione peroxidase (GPx), and heme oxygenase 1 (OH1), thereby protecting cell constituents against oxidative damage [2, 3]. On the other hand, emerging findings indicate that CUR at higher concentrations possesses a prooxidant activity and induces cancer cell apoptosis, thus playing an important regulatory role in cell death during the neoplastic process [4].

As oxidative stress is closely associated with inflammation, there has been a growing interest in determining the anti-inflammatory potential of CUR. Currently, there are a large number of published reports confirming the direct action of CUR on the inflammatory response. Indeed, CUR exhibits a strong anti-inflammatory action by suppression of (i) inflammatory enzymes such as cyclooxygenase 2 (COX2) and lipoxygenase 5 (LOX5), that is, the key enzymes of the arachidonic acid pathways which is involved in the development of human cancer, and inducible nitric oxide synthase (iNOS) that catalyzes the oxidative deamination of l-arginine to produce NO, a potent proinflammatory mediator, (ii) inflammatory cytokines such as tumor necrosis factor *α* (TNF*α*), interleukin- (IL-) 1, 2, 6, 8, and 12, and monocyte chemoattractant protein 1 (MCP1), and (iii) transcription factors like activating protein 1 (AP1) and nuclear factor *κ*B (NF-*κ*B) [5]. The latter is considered not only as one of the most important regulators of proinflammatory gene expression but also as a key player in the development and progression of cancer since its constitutive activation is linked to the proliferation and survival of several types of cancer cells [5].

**Figure 1:**
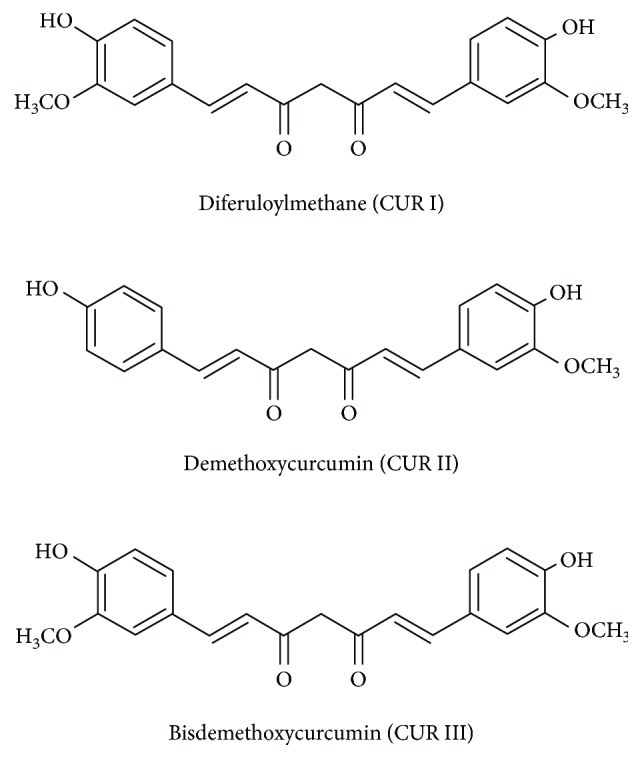
Chemical structures of curcuminoids.

It is noteworthy that commercial CUR contains mixture of three curcuminoids consisting of approx. 77% diferuloylmethane (termed as CUR I), approx. 18% demethoxycurcumin (termed as CUR II), and approx. 5% bisdemethoxycurcumin (termed as CUR III) (Figure 1).

Although health benefits of CUR along with its good tolerance without any toxicity at high oral doses (up to 2.2 g of *Curcuma* extract containing 180 mg of curcumin/day for 4 months) have been demonstrated in patients with advanced refractory colorectal cancer [6], its poor bioavailability due to poor absorption, rapid metabolism, and systemic elimination markedly hampers its clinical application. To overcome these limitations, several different experimental approaches have been employed, including using adjuvants (e.g., piperine, quercetin, and resveratrol) [7] as well as nanoparticle-based delivery systems (e.g., liposomes, solid lipid nanoparticles, niosomes, polymeric nanoparticles, polymeric micelles, cyclodextrins, dendrimers, and silver and gold nanoparticles) [8]. The bioavailability of CUR may also be enhanced through the synthesis of CUR structural analogs [9, 10].

As a significant progress in elucidating the molecular mechanisms underlying antidiabetic and anticancer properties of CUR has been recently achieved, this review will be focused on presenting the molecular targets and pathways engaged in the beneficial effects of CUR.

## 2. Curcumin and T2DM

T2DM is a chronic, multifactorial, metabolic disorder characterized by hyperglycemia as a consequence of insulin deficiency (caused by decreased *β*-cell mass and the dysfunction of existing *β*-cells) and insulin resistance in the liver and peripheral tissues. T2DM is associated with microvascular complications (i.e., nephropathy, retinopathy, and neuropathy) and macrovascular complications (i.e., peripheral vascular disease, cerebrovascular disease, and cardiovascular disease) as well as an increased risk of developing certain types of malignancies, including liver, pancreas, colorectal, bladder, and breast cancers [11].

Obesity, defined as body mass index (BMI) ≥ 30 kg/m^2^, is an important risk factor for the development of T2DM and cardiovascular diseases [12]. The current evidence clearly indicates that weight loss as a result of lifestyle, pharmaceutical, and obesity surgical interventions reduces the risk of developing T2DM in the long term by approximately 32%, 10–74%, and 63%, respectively [13]. Although the pathophysiological mechanisms engaged in the progression of obesity to T2DM are complex and not completely understood, elevated plasma free fatty acids (FFAs), stemming from increased lipolysis in adipose tissue, as well as a low-grade inflammation state and increased oxidative stress have been recognized as the key contributors in this process [12].

The purpose of this section is to present an update on the antidiabetic mechanisms of CUR in various cellular and animal models of T2DM. Moreover, clinical efficacy and safety of CUR for the treatment and prevention of T2DM is described.

It is worth noting that the beneficial effects of CUR have also been proven in various diabetic complications, such as retinopathy, nephropathy, neuropathy, and cardiomyopathy, with the pathogenesis generally linked to hyperglycemia-induced oxidative stress and inflammation. As an overview of the molecular mechanisms targeted by CUR in the aforementioned diabetic complications has been given in several recent publications, this issue is beyond the scope of this review [14].

### 2.1. Molecular Aspects of Antidiabetic Action of CUR in Experimental Diabetic Models

**Figure 2:**
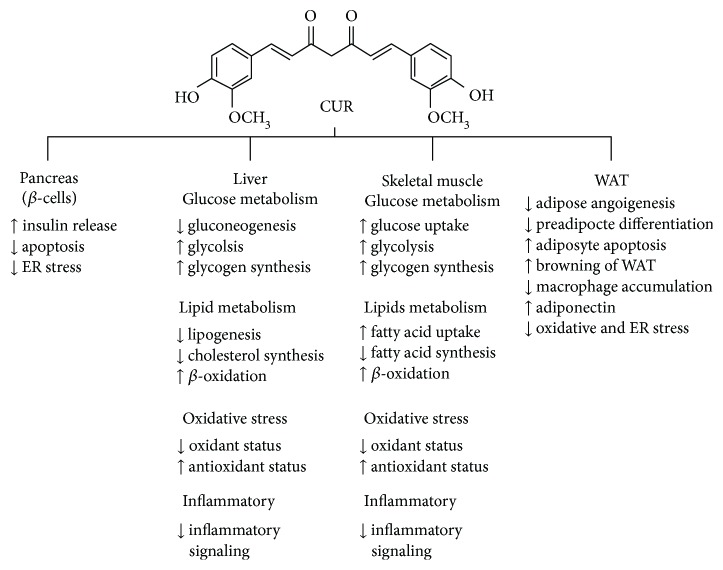
Summary of the antidiabetic effects of CUR in pancreatic *β*-cells, liver, skeletal muscle, and WAT. ↑ increase; ↓ decrease. ER: endoplasmic reticulum; WAT: white adipose tissue.

The structural and functional defects in the insulin-producing and insulin-responsive tissues in the body have been implicated in the pathogenesis of T2DM. It is now generally accepted that CUR ameliorates the pathologic events in these tissues through diverse mechanisms and multiple molecular targets as shown in Figure 2.

#### 2.1.1. Liver

The liver is the major organ responsible for maintaining glucose and lipid homeostasis under physiological conditions. Hence, the dysregulation of hepatic glucose and lipid metabolism may contribute to the development of T2DM. Several lines of evidence from *in vitro* experiments and diabetic animal models highlight a hepatoprotective function of CUR as a consequence of its ability to regulate glucose and lipid metabolism. Indeed, CUR has been shown to suppress hepatic glucose production by activating AMP-activated protein kinase (AMPK), a well-known metabolic stress sensing protein kinase that is activated in response to alterations in cellular energy levels [15, 16]. Furthermore, this polyphenolic compound may also inhibit the key regulatory enzymes for hepatic gluconeogenesis such as glucose-6-phosphatase (G6Pase) and phosphoenolpyruvate carboxykinase (PEPCK) [15]. Some animal studies have revealed that the CUR-mediated hypoglycemic effect may be associated with the enhanced activity of glucokinase (GK), the rate-limiting enzyme for the glycolytic pathway, as well as increased hepatic glycogen storage [17].

Several studies during the past decade have highlighted the key role of CUR in regulating hepatic lipid metabolism. In this context, CUR has been shown to reduce lipogenesis and lipid accumulation in the liver of insulin-resistant rodents, and these effects are associated with the downregulation of the two essential transcription factors involved in hepatic lipogenesis such as the sterol regulatory element-binding protein 1c (SREBP1c) and the carbohydrate response element-binding protein (ChREBP). In addition, CUR positively affects the activity of lipid-regulating enzymes, including fatty acid synthase (FAS), carnitine palmitoyltransferase 1 (CPT1), 3-hydroxy-3-methylglutaryl-coenzyme A reductase (HMGCR), and acyl-CoA:cholesterol acyltransferase (ACAT) [17, 18].

It should be emphasized that CUR also ameliorates chronic liver diseases associated with T2DM, including hepatic steatosis. This disease is characterized by the excessive accumulation of hepatic triglycerides (TGs) resulting from an increased supply of FFAs from peripheral adipose tissue to the liver, enhanced de novo lipid synthesis via the lipogenic pathway, and impaired fatty acid oxidation [19]. There are now strong *in vivo* data supporting the protective role of CUR against hepatic steatosis. Indeed, this compound interferes with the mechanisms involved in abnormal fat accumulation in the liver through the downregulation of a lipogenic transcription factor SREBP1c and its several target enzymes, including acetyl-CoA carboxylase 1 (ACC1) and FAS, and the concomitant upregulation of PPAR*α* via AMPK activation [20]. Another molecular target for CUR in preventing hepatic steatosis is protein-tyrosine phosphatase 1B (PTP1B), a negative regulator of insulin receptor (IR) signal transduction, whose overproduction has been found to associate with defective insulin and leptin signaling in the liver of rats with fructose-induced hepatic steatosis [21]. CUR downregulated hepatic PTP1B with the improvement of insulin and leptin signaling in these animals, as evidenced by enhanced phosphorylation of hepatic IR, insulin receptor substrate 1 (IRS1), and Janus-activated kinase-signal transducer 2 (JAK2) with a concomitant repression of signal transducer and activator of transcription 3 (STAT3) and suppressor of cytokine signaling 3 (SOCS3) and stimulation of protein kinase B (PKB, also known as Akt) and extracellular signal-regulated kinases 1/2 (ERK1/2) [21].

Since oxidative stress has been implicated in the pathogenesis of T2DM through its effect on peripheral tissues and the pancreatic *β*-cell, the hepatoprotective effect of CUR against the oxidative damage has been proven. In animals with experimental T2DM, CUR impaired hyperglycemia-induced oxidative stress through the normalization of the activity of hepatic antioxidant enzymes such as SOD, CAT, and GPx and a reduction of lipid peroxidation, as reflected by the decreased level of the end product of lipid peroxidation—malondialdehyde (MDA) [17].

As oxidative stress is closely linked to the inflammation in T2DM, the anti-inflammatory effects of CUR have also been observed in the liver of insulin-resistant animals, where dietary CUR reduced the activity of NF-*κ*B and several inflammatory markers such as SOCS3, TNF*α*, MCP1, and chemokine (C-C motif) ligand-2 (CCR-2) [22].

#### 2.1.2. Skeletal Muscle

The skeletal muscle is the major site of both glucose disposal and the mitochondrial capacity for energy expenditure, where glucose or lipids is/are used for energy production.

It has been becoming clear that hyperlipidemia, reflected by chronically increased plasma FFAs, leads to an increased uptake of FFAs by the muscle cell and the production of intramuscular fatty acid metabolites (i.e., long-chain acyl-CoA, diacylglycerol, TGs, and ceramides) which stimulate inflammatory cascade and inhibit insulin-stimulated glucose uptake, resulting in the development of skeletal muscles insulin resistance [23]. The cellular mechanisms underlying the accumulation of intracellular lipid intermediates have not been completely elucidated, but lower rates of fatty acid oxidation and higher rates of fatty acid uptake have been proposed as important factors associated with skeletal muscle insulin resistance [23]. CUR has been shown to ameliorate skeletal muscle insulin resistance in rodents with diet-induced insulin resistance via an increase of the uptake and oxidation of fatty acids [24]. These beneficial effects of CUR were strongly linked to the upregulation of fatty acid translocase (CD36), a fatty acid transporter protein implicated in the binding and transport of long-chain fatty acids, and CPT1, the enzyme involved in controlling the entry of long-chain fatty acyl-CoA into mitochondria. CUR also suppressed the ACC activity, a well-known enzyme that catalyzes the ATP-dependent carboxylation of acetyl-CoA to malonyl-CoA during the first step in fatty acid biosynthesis [24]. According to the hypothesis that CUR enhances fatty acid utilization as the energy source, and reduces lipid metabolites accumulation, Seo et al. [17] reported that CUR supplementation increases the skeletal muscle lipoprotein lipase (LPL) activity in insulin-resistant rodents. Currently, it is well established that LPL plays a rate-limiting role in TG-rich lipoprotein metabolism and its activity is associated with skeletal muscle insulin sensitivity [25].

In addition to weakening lipotoxicity in skeletal muscle, CUR may also exert beneficial effect on insulin-induced glucose transport in this tissue via the glucose transporter (GLUT) 4 system. It is known that the insulin-stimulated glucose uptake is impaired in patients with T2DM as a consequence of inhibiting GLUT 4 translocation to the cell membrane [26].

CUR has been shown to improve insulin sensitivity in muscle tissue of insulin-resistant rodents by an *increase* of the cellular glucose uptake as a result of the enhancement of GLUT4 *translocation* from intracellular compartments to the plasma membrane [24, 27]. In addition, the increased glucose oxidation and glycogen synthesis has also been found in the muscle cell [24, 27]. Importantly, the regulatory effect of CUR on glucose and lipid metabolism in skeletal muscle was mediated by the liver kinase B1- (LKB1-) AMPK signaling pathway, suggesting that AMPK might serve as a potential target of CUR to enhance insulin sensitivity in skeletal muscle [24, 28].

Compelling evidence indicates that a defect in the insulin signaling pathway at the IRS1 level is the primary post receptor abnormality resulting in skeletal muscle insulin resistance. The positive effect of CUR on the insulin signaling cascade by enhancing IRS1 tyrosine phosphorylation has been observed in insulin-resistant rodents [29]. In this study, CUR also attenuated the inflammatory signaling through suppression of NF-*κ*B, COX2, and protein kinase C-theta (PKC*θ*). Furthermore, CUR decreased the oxidant status and increased the antioxidant status in the skeletal muscle, as evidenced by the reduced levels of MDA and total oxidant status (TOC) and increased muscle GPx expression. The latter effect was associated with impaired ERK1/2 and p38 activation [29]. Overall, these results indicate that CUR ameliorates skeletal muscle pathology via glucose and lipid metabolic changes as well as the improvement in antioxidant defense mechanism and anti-inflammatory effects.

#### 2.1.3. Adipose Tissue

Adipose tissue is not only the main site of lipid storage but also an endocrine organ responsible for the synthesis and secretion of various bioactive molecules, collectively called adipokines or adipocytokines. Accumulating evidence indicates that adipose tissue dysfunction, characterized by abnormal adipokine production, increased FFA release and inflammation, plays an important role in the pathogenesis of T2DM [30]. As the expansion of adipose tissue leads to inflammatory and endoplasmatic reticulum (ER) stress responses, some studies have suggested that CUR might hold protective or ameliorating effects against these pathophysiologic events through its anti-inflammatory and antioxidative properties. In this regard, beneficial effects of CUR have been shown to associate with reduced macrophage infiltration of adipose tissue in obese mice. Furthermore, CUR improved the adipose tissue production of the anti-inflammatory adipokine adiponectin and several stress response proteins, including Sirtuin 1 (Sirt1), heat shock proteins 70 and 90 (Hsp70 and Hsp90), and forkhead transcription factor FKHR (Foxo1) [22]. Other studies in this area have confirmed the positive effect of CUR on adipose tissue inflammation by repressing several important mediators of the inflammatory response in adipocytes, including IL-1*β*, IL-6, TNF*α*, MCP1, and COX2. This protection effect of CUR stemmed from an inhibition of the NF-*κ*B signaling pathway through preventing I*κ*B degradation and, in turn, reducing NF-*κ*B nuclear translocation in adipocytes [31].

It is well established that the development of obesity-related diseases is a result of the expansion of adipose tissue and the formation of new blood vessels for delivering oxygen and nutrients to adipocytes [32]. Thus, controlling fat mass is tightly associated with the modulation of both adipogenesis and angiogenesis. Several *in vitro* studies have found the antiadipogenic activity of CUR in 3T3-L1 cells, and AMPK as well as the canonical Wnt/*β*-catenin signaling and its downstream targets such as c-Myc and cyclin D1 have been implicated in this process [33, 34].

The hypothesis that blocking adipose tissue angiogenesis with CUR is a successful strategy for the therapy of obesity-related diseases has been proven by some studies. In fact, CUR inhibits adipose tissue neovascularization through suppression of vascular endothelial growth factor (VEGF) and its receptor VEGFR2 (i.e., the two major angiogenic factors involved in the stimulation of proliferation and migration of endothelial cells) and the stimulation of adipocyte energy metabolism, predominantly by AMPK activation [33].

In recent years, many studies have also focused on CUR as the so-called browning agent of adipose tissue that enhances heat production, thereby resulting in slimming. In this regard, CUR has been reported to induce the development of beige adipocytes in inguinal white adipose tissue (WAT) through the norepinephrine-*β*3 androgen receptor (AR) pathway, as reflected by beige cells emerging in inguinal WAT, and the increased expression of a panel of brown fat-specific genes in inguinal WAT [35].

#### 2.1.4. Pancreatic *β*-Cells

Pancreatic *β*-cells are metabolic sensors which adjust insulin secretion to glucose levels in the bloodstream under physiological conditions. In subjects with T2DM, these cells are unable to produce a sufficient amount of insulin required for blood glucose control. In recent years, several studies have emphasized the importance of CUR in the enhancement of pancreatic *β*-cell function under diabetic conditions. In this context, Best et al. [36] demonstrated the stimulatory effect of CUR on *β*-cells that was associated with the depolarization of the cell membrane potential, the generation of electrical activity, and an increase in insulin release from these cells. The authors concluded that the aforementioned consecutive events induced by CUR may contribute to the enhancement of insulin action and subsequently a decrease of the blood glucose levels. Another proposed mechanism by which CUR may improve insulin secretion in islets includes the downregulation of cyclic nucleotide phosphodiesterases (PDEs), that is, the enzymes catalyzing the conversion of cAMP and cGMP into 5′-AMP and 5′-GMP, respectively. Thus, CUR may participate in the regulation of the cellular levels of the second messengers, and therefore, this compound should be considered as a PDE inhibitor to improve pancreatic *β*-cell function [37].

Other cellular mechanisms underlying the beneficial CUR action on *β*-cells have been proposed, including suppression of cell apoptosis, improvement of glucose-induced insulin secretory function by Akt activation, inhibition of nuclear translocation of Foxo1, impairment of ER stress, and promotion of mitochondrial survival pathway under elevated FFAs, especially the saturated FFAs such as palmitate [38].

### 2.2. The Effect of CUR on Metabolic Disturbances in Animal and Human Studies

#### 2.2.1. Animal Models

In the last decade, antidiabetic properties of CUR have been extensively studied in various animal models of obesity (genetically and diet-induced) and T2DM (Table 1). The ability of this compound to improve glycemic status and insulin sensitivity has been demonstrated in high-fat-diet- (HFD-) fed C57BL/6J mice and C57BL/6J *ob*/*ob* mice with a spontaneous knockout mutation of the leptin gene that were treated with CUR-supplemented diet [18, 22, 39]. The insulin-sensitive action of CUR in these diabetic animals involved the improvement of insulin signaling in adipose tissue and hepatocytes as well as the attenuation of lipogenesis in the liver and the oxidative stress and inflammatory processes in adipose tissue. In addition, CUR supplementation was associated with decreased body weight or fat composition in HFD-fed mice [18, 22, 39]. As rosiglitazone is the antihyperglycemic drug that acts through the activation of peroxisome proliferator-activated receptor *γ* (PPAR*γ*), that is, a number of the nuclear hormone receptor superfamily that regulates the transcription of several genes involved in adipocyte differentiation, glucose and lipid metabolism, and inflammation [40], its antidiabetic efficiency was compared to that of CUR in rodents with diet-induced insulin resistance [41]. Interestingly, both compounds exhibited a similar improvement in glucose tolerance, lipid profile, and insulin sensitivity. The latter effect of CUR was partially attributed to its anti-inflammatory action, as reflected by the reduced plasma TNF*α* levels as well as its antilipolytic action, as evidenced by decreased plasma FFA levels [41]. More recently, the positive effects of CUR on metabolic abnormalities associated with T2DM have been confirmed in high-fructose-fed Wistar rats, where CUR treatment attenuates insulin resistance and glucose intolerance as a consequence of its antioxidative and anti-inflammatory actions [29].

The beneficial effects of CUR have also been observed in other diabetic animal models such as KK-A^y^ mice, in which CUR reduced hyperglycemia through PPAR*γ* activation [42], and C57BL/KsJ-*db*/*db* mice, in which CUR reduced insulin resistance and hyperglycemia as a result of the increased blood insulin concentration that subsequently leads to enhanced glycolysis and impaired gluconeogenesis in the liver and augmented LPL activity in skeletal muscle [17]. Of note, CUR was also able to reduce a hyperglycemia-induced oxidative stress in erythrocytes and the liver of *db*/*db* mice, pointing to its protective role against diabetic complications.

Taken together, animal studies are generally consistent in respect to the beneficial effect of CUR on insulin action in the liver, skeletal muscle, and adipose tissue in various models of insulin resistance.

#### 2.2.2. Studies in Humans

Clinical implications of CUR, used in different doses and forms, have been demonstrated by the results obtained from only few small studies involving individuals with T2DM. In general, these studies have confirmed the beneficial effects of CUR on some metabolic and inflammatory indices in diabetic patients. In fact, curcuminoids supplementation (300 mg/day for 3 months) in overweigh/obese T2DM patients reduced the plasma levels of fasting blood glucose (FBG), glycosylated hemoglobin (HbA_1c_), FFAs, TGs, C-reactive protein (CRP), TNF*α*, IL-6, and adipocyte fatty acid-binding protein (A-FABP, also termed FABP4). The latter is a member of the cytosolic fatty acid-binding protein family that is highly produced by adipocytes and participates in intracellular fatty acid trafficking. In T2DM patients supplemented with curcuminoids, the plasma A-FABP level positively correlated with blood glucose, FFAs, and CRP levels, implying that a decrease in the plasma A-FABP level may be associated with the improvement of metabolic and inflammatory indices in diabetic subjects. Furthermore, curcuminoids increased plasma SOD activity and decreased insulin resistance index (HOMA-IR) in T2DM subjects [43, 44]. More recently, the efficacy of nano-CUR (as nano-micelle 80 mg/day for 3 months) on glucose and lipid parameters has been demonstrated in T2DM subjects, as evidenced by the decreased levels of HbA_1c_, glucose, and low-density lipoprotein cholesterol (LDL-C) and reduced body mass index (BMI) [45]. Of note, there is only a single randomized, double-blind, placebo-controlled clinical trial on the evaluation of a protective role of CUR against T2DM development in a prediabetic population, referring to individuals with elevated blood glucose levels but do not meet diagnostic criteria for T2DM. In this study, CUR supplementation was rather safe for prediabetic patients, leading to improved *β*-cell function, as reflected by an increase in homeostasis model of assessment for *β*-cell function (HOMA-B), reduction in the C-peptide level and HOMA-IR, and increased adiponectin level [46]. Adiponectin (AdipoQ), also known as adipocyte complement-related protein of 30 kDa (Acrp30) and gelatin-binding protein of 28 kDa (GBP28), is a well-known adipokine with insulin-sensitizing effect. Although the molecular mechanism(s) by which AdipoQ may impair insulin resistance is not completely clear, its role in the stimulation of fatty acid oxidation in the liver and muscle, activation of glucose uptake in muscle, and suppression of gluconeogenesis in the liver has been well documented [47].

## 3. Curcumin and Cancer

Cancer is a complex and multistep process that develops as a result of the accumulation of genetic and epigenetic alterations. These changes lead to sustaining proliferative signaling, evading growth suppressors, resisting cell death, enabling replicative immortality, inducing angiogenesis, and activating invasion and metastasis [48]. All these pathological events are linked to the coordinated action of numerous signaling pathways, creating a very complex but not yet fully understood intracellular picture of various networks associated with the development and progression of cancer. Knowledge about alterations in the expression and function of key components of these signal transduction pathways provides potential targets for anticancer therapy. Despite the introduction of approx. 150 anticancer cytotoxic and targeted drugs into clinics, the current treatment of cancers remains still far from satisfactory [49, 50]. Hence, there is imperative to develop safe and effective anticancer therapeutic strategies.

The chemotherapeutic potential of CUR has been shown in various types of cancers (i.e., multiple myeloma, lung, prostate, breast, ovarian, bladder, liver, gastrointestinal tract, pancreatic, colorectal epithelial cancer, and lymphomas) as a consequence of its inhibitory action on multiple carcinogenic signaling pathways. Although the exact molecular mechanisms underlying the anticancer CUR action remain unclear so far, multiple cellular targets for this polyphenol have been identified in numerous cancer model systems. Among them are the following: (i) transcription factors (e.g., AP1; STAT3; NF-*κ*B; hypoxia-inducible factor 1*α*, HIF1*α*; nuclear factor erythroid 2- (NF-E2-) related factor, Nrf2; peroxisome proliferator-activated receptor *γ*, PPAR*γ*), (ii) growth factors (e.g., fibroblast growth factor 2, FGF2; hepatocyte growth factor, HGF; platelet-derived growth factor, PDGF; VEGF; transforming growth factor *β*, TGF*β*; angiopoietin 1 and 2, Ang 1 and 2), (iii) receptors (e.g., IL-8 receptor, IL-8R; epidermal growth factor receptor, EGFR), (iv) kinases (e.g., ERK; mitogen-activated protein kinase (MAPK); protein kinase A, PKA; PKB/Akt; PKC), (v) cytokines (e.g., TNF*α*; IL-1, -2, -5, -6, -8, -12, and -18; MCP1), (vi) enzymes other than kinases (e.g., COX2; iNOS; telomerase; matrix metalloproteinases, MMPs), and (vii) adhesion molecules (e.g., endothelial leukocyte adhesion molecule 1, ECAM1; intracellular adhesion molecule 1, ICAM1; vascular cell adhesion molecule 1, VCAM1). A detailed review of the entire list of intracellular biomolecules targeted by CUR has been recently published by Kunnumakkara et al. [51].

This section was designed to highlight the anticancer effects of CUR associated with its ability to modulate the activity of key factors in cellular signal transduction pathways which are involved in the initiation, promotion, and progression of tumors.

### 3.1. Proapoptotic Effects of CUR

Apoptosis is a tightly regulated process responsible for maintaining tissue homeostasis in multicellular organisms, and its functional disturbances contribute to oncogenic transformation and therapeutic resistance. Caspases are critical executioners of the apoptotic process consisted of the two major signaling pathways such as the intrinsic (i.e., mitochondria-dependent apoptosis) and extrinsic (i.e., mitochondria-independent apoptosis induced by death receptor activation) pathways. The latter is mediated through FasL and Fas/CD95 receptors followed by caspase 8 activation whereas the mitochondrial pathway is mediated through the change in the ratio of Bcl2-associated X (Bax)/B-cell lymphoma 2 (Bcl2) proteins, loss of mitochondrial membrane potential and then by caspase 9 activation, which leads to caspase 3 activation and ultimately nuclear DNA fragmentation [52].

In recent years, the proapoptotic effect of CUR has been documented in a variety of cancer cell lines and *in vivo* animal studies (Table 2). For example, CUR induced apoptosis via the upregulation of the proapoptotic Bax and the downregulation of the antiapoptotic proteins such as myeloid cell leukemia 1 (Mcl1) and Bcl2 in human melanoma cells. In addition, CUR activated caspase 3 and caspase 8 and deregulated the expression of several apoptosis-associated proteins such as NF-*κ*B, p38 MAPK, and p53 [53]. In human nonsmall cell lung cancer, CUR stimulated the mitochondrial apoptosis pathway by reducing the mitochondrial membrane potential in a dose-dependent manner and subsequently releasing cytochrome *c* from mitochondria to the cytoplasm as a result of the Bax upregulation and the Bcl2 downregulation [54]. A recent *in vitro* model of lung cancer has been provided evidence for CUR-mediated apoptosis through the activation of the p53-miR192-5p/215/X-linked inhibitor of apoptosis (XIAP) pathway, suggesting a regulatory role of CUR as an epigenetic agent for miRNAs in suppression of human lung cancer [55]. The miRNAs are short and highly conserved noncoding RNAs involved in multiple steps in carcinogenesis, and miR-192-5p/215 have been recognized as putative tumor suppressors in nonsmall cell lung cancer [55]. The epigenetic activity of CUR on miRNAs has also been confirmed in MCF7 breast cancer cells, where CUR upregulated miRNA15 and miRNA16 and this effect was associated with the Bcl2 downregulation and apoptosis induction in these cells [56]. Furthermore, in MDA-MB-435 breast cancer cells, CUR suppressed the expression of histone methyltransferase, which is typically overexpressed in human breast cancers with poor prognosis, through the stimulation of the MAPK pathway, and this event was accompanied by G1 cell cycle arrest [57]. Of note, there is convincing evidence that there are other mechanisms underlying the growth inhibitory activity of CUR against human breast cancer, including suppression of the microtubule assembly dynamics linked to the activation of the mitotic checkpoint [58] and the Wnt/*β*-catenin signaling pathway that is an important regulator of tumor cell invasion and metastasis [59].

The oncostatic action of CUR on neoplastic cells may also result from its capability of stimulating ROS production in various malignant cancer cell lines. In support of this, Li et al. [60] demonstrated that CUR-mediated ROS overproduction in liver cancer cells upregulates the toll-like receptor 4 (TLR4), that is, a key player in the inflammatory process, which subsequently stimulates the myeloid differentiation primary response protein MyD88, a downstream adaptor molecule of TLR4, leading to apoptosis [60]. The relationship between CUR-mediated ROS generation and apoptosis was further confirmed in gastric cancer cells (where ROS induced the apoptosis signal-regulating kinase 1 (ASK1)/MAPK kinase (MKK) 4/c-Jun N-terminal kinase (JNK) signaling pathway) [61] and osteosarcoma cells (where ROS induced the mitochondrial cytochrome *c*/caspase 3 apoptotic pathway) [62].

The proapoptotic effect of CUR has also been largely attributed to its modulatory effect on the transcriptional factor NF-*κ*B that is constitutively overexpressed in cancer. In melanoma cells, CUR inhibited NF-*κ*B in a dose-dependent manner and, in turn, induced apoptosis [63]. More recently, Cao et al. [64] showed that CUR-mediated NF-*κ*B suppression in giant cell tumor of bone leads to the inhibition of cell proliferation and promotion of apoptosis through the stimulation of the JNK signaling pathway.

*In vitro* studies have also proved that CUR may induce apoptosis in acute myeloid leukemia cells via a simultaneous attenuation of the two critical prosurvival signaling pathways such as the Akt/mammalian target of rapamycin (mTOR) pathway and the Raf/mitogen-activated signal-regulated protein kinase (MEK)/ERK pathway [65].

It is noteworthy that although many studies have been focused on the proapoptotic properties of CUR, other types of CUR-mediated cell death pathways, including autophagy, mitosis catastrophe, and paraptosis, have also been recognized in some experimental studies [66].

### 3.2. Anti-Invasion Effects of CUR

Several lines of evidence indicate that CUR does not only affect the tumor growth, but also inhibits the tumor development, primarily by suppressing MMPs, that is, zinc-dependent endopeptidases participating in the excessive degradation of the extracellular matrix (ECM) during tumor invasion and metastasis (Table 2). Indeed, CUR has been shown to repress the migration and invasion of tongue carcinoma cells through the downregulation of MMP10, which is usually increased in this type of cancer [67]. In breast cancer cells, CUR downregulated MMP9 by blocking the PKC*α*/MAPK/NF-*κ*B/AP1 pathway (in the MCF7 cell line) or the TGF*β*/Smad and TGF*β*/ERK signaling pathways (in the MDA-MB-231 cell line) [68, 69]. CUR also downregulated MMP2/9 in endometrial carcinoma cells via suppressing the ERK signaling pathway [70]. Recently, the novel antimetastatic mechanism of CUR action in colon cancer has been proposed [71]. According to this, CUR can reduce the level of the active phosphorylated form of cortactin (i.e., the pTyr421), overexpressed in colon cancer cells, through a direct physical interaction with the nonreceptor type 1 protein-tyrosine phosphatase (PTPN1), leading to an increased PTPN1 activity and subsequently reduced cancer cell migration. Although the role of the PTPN1/cortactin axis has been identified in the mechanism of CUR-induced inhibition of colon cancer cell migration, additional studies are required to establish the mechanism by which CUR activates PTPN1. Moreover, verifying the significance of the PTPN1/cortactin pathway in cell metastasis of other cancers is needed.

### 3.3. Chemosensitizing Properties of CUR

An important aspect of the anticancer activity of CUR is its ability to sensitize cancer cells to chemotherapeutics, thereby enhancing the anticancer effect of chemotherapeutics and improves the efficacy of cancer treatment.

The synergistic effect of the combination of CUR with different anticancer drugs used widely in clinical practice (e.g., cisplatin, carboplatin, 5-fluorouracil (5-FU), gemcitabine, and paclitaxel) has been observed in various cancer types, including lung, breast, colon, pancreas, gastric, liver and prostate cancers [72]. Although the mechanism of CUR action as a chemosensitizer involves multiple targets participating in the survival signaling pathways, the transcription factor NF-*κ*B appears to be one of crucial biomolecules targeting by CUR [72]. The phase II clinical trial has revealed that CUR intake (8 g per day) causes the NF-*κ*B downregulation in peripheral blood mononuclear cells of patients with advanced pancreatic cancer and, moreover, the combination treatment of CUR with gemcitabine is safe in these patients [73]. In another related human study, an oral intake of CUR (8 g per day) in combination with gemcitabine-based chemotherapy resulted in a median survival time of 161 days and a 1-year survival rate of 19% for patients with pancreatic cancer, suggesting that CUR may potentiate traditional chemotherapeutic agent [74]. A good potential of CUR has also been found in combination with standard docetaxel chemotherapy in patients with advanced breast cancer and castration-resistant prostate cancer [75, 76]. According to http://www.clinicaltrials.gov, there are presently several interventional studies in various stages evaluating CUR as an adjunctive treatment for some cancer types. In these studies, CUR is used in combination with the following anticancer agents: (i) 5-FU in patients with chemoresistant metastatic colorectal cancer (NCT02724202; *n* = 14), (ii) tyrosine kinase inhibitors such as gefitinib (Iressa) and erlotinib (Tarceva) in patients with EGFR-mutant advanced nonsmall cell lung cancer (NCT02321293; *n* = 20), (iii) docetaxel in patients with HER2 negative locally advanced or metastatic breast cancer (NCT00852332; *n* = 100), (iv) gemcitabine along with paclitaxel and metformin in patients with unresectable pancreatic cancer (NCT02336087; *n* = 21), and (v) irinotecan in patients with metastatic adenocarcinoma of the colon or rectum (NCT01859858; *n* = 23). These clinical trials are primarily concentrated on determining the feasibility, safety, and efficacy of CUR used in combination with the aforementioned anticancer agents. Of note, the effect of CUR on irinotecan pharmacokinetics was also investigated to better understand the interaction between CUR and the anticancer drug. Taken together, the findings obtained from these studies might lead to improved dosing guidelines and more effective treatment of different types of cancer with less toxicity.

### 3.4. Radiosensitizing Properties of CUR

CUR has also proven to be a radiosensitizer for cancer cells by the downregulation of different growth regulatory pathways and the protection of normal cells from radiotherapy-induced toxicity [77]. Apart from the ability of CUR to enhance the effect of radiation treatment on cancer cells, CUR also possesses the radioprotective properties. Recently, the double-blind, placebo-controlled clinical trial has provided the data supporting the radioprotective effect of CUR in patients with prostate cancer treated with radiotherapy. In this study, CUR supplementation (3 g per day) impaired the severity of radiotherapy related with urinary symptoms in patients with prostate cancer, thus improving their quality of life [78]. The radioprotective effect of CUR has also been observed in another randomized, double-blind, placebo-controlled clinical trial, where oral CUR (6 g per day) reduced radiation dermatitis severity in breast cancer patients receiving radiotherapy [79]. As radiation-induced dermatitis occurs in approx. 95% of patients receiving radiotherapy for breast cancer and, moreover, as there is no standard treatment for the prevention of radiation-induced dermatitis, CUR appears to be an effective protector against radiation-induced skin reactions in breast cancer patients receiving radiotherapy. However, due to a small group of the participants, further clinical trials with large groups of patients should be performed to confirm the long-term safety and effectiveness of CUR treatment in the radiotherapeutic strategy for breast cancer patients. Additionally, more molecular research is needed to identify the biological mechanisms involved in the radioprotective effect of CUR in breast cancer. It should be emphasized that there is also an ongoing randomized clinical trial (NCT00745134; http://www.clinicaltrials.gov) focused on determining whether the combination of CUR with standard radiotherapy and chemotherapy (i.e., capecitabine) is able to shrink or slow the growth of rectal cancer as well as reduce some of the side effects of the radio- and chemotherapeutic approaches.

### 3.5. Nanoformulations of CUR

The low solubility and poor bioavailability of CUR, mainly due to intestinal and hepatic glucuronidation, limit its application as a therapeutic agent in clinical oncology. In 2004, Garcea et al. [80] reported that CUR consumption at a dosage of 450–3600 mg per day results in undetectable plasma CUR levels in patients with hepatic metastases from colorectal cancer. To overcome this issue and improve CUR pharmacokinetic properties and its anticancer therapeutic potential, various novel delivery systems have been developed in recent years, including liposomes, solid lipid nanoparticles, niosomes, polymeric nanoparticles, polymeric micelles, cyclodextrins, dendrimers, and silver and gold nanoparticles. A comprehensive characteristic of CUR nanoformulations in respect to their beneficial effects in drug delivery has been recently presented by Mehanny et al. [8] and is beyond the scope of this review. However, it should be highlighted that among numerous advanced drug delivery strategies for CUR, poly(d,l-lactic-co-glycolic acid) (PLGA) is currently one of the most commonly used polymers due its nontoxic, biodegradable, and nonimmunogenic nature. Furthermore, the nano-CUR-loaded PLGA has been shown to exhibit enhanced bioavailability up to 22-fold compared to conventional CUR in an experimental animal model [81]. More recently, CUR conjugated with PLGA has been reported to possess an improved sustainability, cellular uptake, and efficiency in suppressing cell proliferation and inducing apoptotic cell death signaling in human colon carcinoma cells compared to native CUR [82]. The enhanced anticancer efficacy of a PLGA-CUR formulation is in accordance with other studies demonstrating a higher effectiveness of PLGA-CUR than free CUR in repressing prostate cancer cell growth under *in vitro* and *in vivo* conditions. The mode of action of PLGA-CUR comprised the inhibition of cellular signaling molecules such as Akt and STAT3 and, in turn, the induction of apoptosis via the downregulation of the antiapoptotic proteins such as Bcl-xL and Mcl1 and the stimulation of the poly (ADP-ribose) polymerase (PARP) cleavage. In prostate cancer cells, PLGA-CUR also suppressed of the expression of AR and nuclear *β*-catenin. The latter is the major downstream effector of the canonical Wnt pathway and the AR coactivator in prostate cancer development. Furthermore, PLGA-CUR downregulated miR21 and upregulated miR205; thus, both miRNAs involved in prostate cancer progression and metastasis (Table 2) [83].

## 4. Conclusion

CUR is one of the most commonly studied natural compounds in respect to T2DM and cancer. A large body of evidence from preclinical studies indicates that CUR possesses a number of antidiabetic health benefits via attenuating hyperglycemia, hyperlipidemia, and insulin resistance. These positive effects of CUR appear to predominantly result from its pleiotropic effects on diverse molecular mechanisms associated with abnormal glucose and lipid metabolism, inflammation, and oxidative stress; thus, all the pathological changes occurred during the development and progression of T2DM. Of note, the promising antidiabetic effects of CUR found in cellular and animal models were also confirmed in few small clinical studies. Despite encouraging results, additionally large-scale, well-controlled trials regarding doses, effectiveness, and safety of CUR remain to be conducted.

In recent years, a significant research effort has also been focused on elucidating the signaling pathways involved in the oncostatic action of CUR. A great deal of experimental data obtained from *in vitro* and *in vivo* models of carcinogenesis support CUR as an effective regulator of cancer promotion and progression. The anticancer-promoting effects of CUR have largely been attributed to its ability to suppress cell growth, angiogenesis, and metastasis as well as to induce apoptosis in a variety of cancer types. Furthermore, the positive effects of the combined treatment of CUR with conventional chemotherapy drugs or radiotherapy should be considered as a subject of subsequent research, opening up new opportunities for effective intervention in cancer treatment. In spite of the beneficial biological activities of CUR, its poor bioavailability markedly limits its clinical application. To overcome this issue, various nanotechnology-based drug delivery systems have been developed and investigated *in vitro* and *in vivo*, giving satisfactory results. Importantly, CUR is safe and well tolerated in human clinical trials of cancer.

## Figures and Tables

**Table 1:** The antidiabetic efficacy of CUR in animal models of the disease.

Animal model	Dose/duration	Cellular target/pathway	Effect	References
KK-A^y^ mice	0.1 or 0.5 g of CUR/100 g of diet for 4 weeks; 0.2 or 1.0 g of CUR/100 g of diet for 4 weeks	↑ adipocyte differentiation ➔ PPAR*γ*	↓ blood glucose	[42]

C57BL/KsJ-*db*/*db* mice	0.2% CUR in diet for 6 weeks	↓ hepatic gluconeogenic enzymes (G6Pase, PEPCK), hepatic lipid-regulating enzymes (FAS, CPT, HMGCR, ACAT), hepatic MDA ↑ hepatic glycolytic enzyme GK, glycogen content, and antioxidant enzymes (CAT, GPx, SOD), skeletal muscle LPL	↓ blood glucose, HbA_1c_, HOMA-IR, FFAs, TGs, TC	[17]

C57BL/6J mice	CUR (50 or 100 mg/kg/d)	↑ browning of WAT, thermogenic gene expression, mitochondrial biogenesis, plasma norepinephrine level ➔ *β*3AR gene expression in WAT	↓ body weight and fat mass ↑ cold tolerance	[35]

C57BL/6J mice on HFD; C57BL/6J *ob*/*ob* mice	0.5% CUR in diet for 6 weeks	↑ adipose tissue adiponectin production, adipose tissue expression of stress response genes (*Sirt1*, *Hsp70* and *Hsp90*, *Foxo1*) ↓ macrophage infiltration of WAT, hepatic NF-*κ*B, markers of hepatic inflammation	↓ blood glucose, HbA_1c_, insulin resistance, body weight	[22]

C57BL/6J mice on HFD	HFD with CUR (4 g/kg diet) 2 days per 1 week	↑ insulin stimulated PKB/Akt Ser473 phosphorylation in adipose tissue and hepatocytes, adipocyte HO1 ↓ hepatic NF-*κ*B, hepatic lipogenic transcription factors (SREBP1c, ChREBP), inflammatory markers in mature adipocytes	↓ blood glucose, insulin resistance, liver weight, intrahepatic lipid content, body weight	[18]
	HFD with CUR (40 and 80 mg/kg/d) for 12 weeks	↑ UCP1, LPL, adiponectin in BAT and WAT ↓ lipid-regulating enzymes (FAS, SCD1, HMGCR) in BAT and WAT, hepatic gluconeogenic enzymes (G6Pase, PEPCK), hepatic lipid-regulating transcription factors and enzymes (SREBP1 and 2, FAS, SCD1, HMGCR)	↓ serum FBG, insulin, TC, TGs, and LDL-C, insulin resistance, liver weight, hepatic TC and TGs, body weight, epididymal fat weight, adipocyte diameter, size of brown adipocytes	[39]
	HFD with CUR (0.15%) for 11 weeks	↑ hepatic PPAR*α*, AMPK ↓ hepatic SREBP1, ACC1, FAS, CD36	↓ serum TC, TGs, FBG, and insulin, HOMA-IR, hepatic lipid accumulation, body weight, visceral adipose tissue	[20]
	CUR (500 mg/kg) for 12 weeks	↓ VEGF*α*, PPAR*γ*, C/EBP*α*	↓ body weight gain, liver weight, microvessel density, serum TC, FBG, TGs and FFAs tendency --| adipokine-induced angiogenesis	[33]

Sprague Dawley rats on HFD	CUR (80 mg/kg), rosiglitazone (1 mg/kg), and their combination for 15 days	↓ TNF*α*, lipolysis	↓ serum, TC, TGs, LDL-C, FFAs, FBG, and insulin, insulin resistance ↑ HDL-C	[41]

Sprague Dawley rats on high-fructose diet	CUR (15–60 mg/kg) for 4 weeks	↑ hepatic IR, IRS1, JAK2 ↓ hepatic PTP1B, SOCS3, STAT3 ➔ hepatic Akt, ERK1/2	↓ serum TGs, VLDL, TNF*α*, leptin, and insulin, insulin resistance, hepatic TGs, TC and VLDL	[21]

Wistar rats on HFD + STZ	CUR (50–200 mg/kg) for 7 weeks	↑ skeletal muscle CD36, CPT1, ↓ PDK4, GS phosphorylation	↓ serum FFAs, TGs, TC, FBG, insulin resistance ↑ fatty acids uptake and oxidation, glucose oxidation, glycogen synthesis	[24]

Wistar rats on high-fructose diet	CUR (200 mg/kg) for 10 weeks	↑ skeletal muscle IRS1 tyrosine phosphorylation, GPx ↓ skeletal muscle TG, MDA, TOS, ERK1/2, p38 MAPK, COX2, PKC*θ*, NF-*κ*B activation	↓ serum insulin, TNF*α*, CRP, and FBG, insulin resistance ↑ serum adiponectin	[29]

➔ induction; --| inhibition; ↑ increase; ↓ decrease. ACAT: acyl-CoA:cholesterol acyltransferase; ACC1: acetyl-CoA carboxylase 1; *β*3AR: *β*3-adrenergic receptor; BAT: brown adipose tissue; CAT: catalase; CD36: fatty acid translocase; ChREBP: carbohydrate response element-binding protein; COX2: cyclooxygenase 2; CPT1: carnitine palmitoyltransferase 1; CRP: C-reactive protein; ERK1/2: extracellular signal-regulated protein kinases 1 and 2; FAS: fatty acid synthase; FBG: fasting blood glucose; FFA: free fatty acid; GK: glucokinase; G6Pase: glucose-6-phosphatase; GPx: glutathione peroxidase; GR: glutathione reductase; GS: glycogen synthase; GSH: reduced glutathione; HbA_1c_: glycosylated hemoglobin; HDL: high-density lipoprotein cholesterol; HFD: high-fat diet; HO1: heme oxygenase 1; HOMA-IR: homeostatic index of insulin resistance; HMGCR: 3-hydroxy-3-methylglutaryl-coenzyme A reductase; Hsp70 and Hsp90: heat shock proteins 70 and 90; IR: insulin receptor; IRS1: insulin receptor substrate 1; JAK2: Janus-activated kinase-signal transducer 2; LDL-C: low-density lipoprotein cholesterol; LPL: lipoprotein lipase; MAPK: mitogen-activated protein kinase; MDA: malondialdehyde; PDK4: pyruvate dehydrogenase kinase 4; PEPCK: phosphoenolpyruvate carboxykinase; PKB/Akt: protein kinase B or Akt; PKC*θ*: protein kinase C-theta; PPAR*α* and *γ*: peroxisome proliferator-activated receptor *α* and *γ*; PTP1B: protein-tyrosine phosphatase 1B; SCD1: stearoyl-coenzyme A desaturase 1; Sirt1: Sirtuin 1; SOCS3: suppressor of cytokine signaling 3; SREBP1c: sterol regulatory element-binding protein 1c; STAT3: signal transducer and activator of transcription 3; STZ: streptozotocin; TC: total cholesterol; TG: triglyceride; TNF*α*: tumor necrosis factor *α*; TOS: total oxidant status; UCP1: uncoupling protein 1; VEGF: vascular endothelial growth factor; WAT: white adipose tissue.

**Table 2:** Overview of anticancer action of CUR in selected cellular models.

CUR	Human cell line/animal model	Dose/duration	Cellular target/pathway	Effect	References
CUR	Melanoma cell lines A375, MV3, and M14	30 *μ*M for 24 h	↑ Bax, caspases 8 and 9 ↓ Mcl1, Bcl2	--| cell proliferation ➔ apoptosis	[53]
	Nonsmall cell lung cancer cell line A549	40 *μ*M for 24 h	↑ Bax, cytochrome *c* release ↓ Bcl2, the mitochondrial membrane potential	--| cell proliferation ➔ apoptosis	[54]
	Nonsmall cell lung cancer cell lines H460 and A427	40 *μ*M for 24 h	➔ p53-miR192-5p/215-XIAP pathway	--| cell proliferation ➔ apoptosis	[55]
	Liver cancer cell line MHCC97H	60 *μ*M for 24 h	↑ ROS ➔ TLR4/MyD88/caspase 8/caspase 3 pathway	--| cell proliferation ➔ apoptosis	[60]
	Gastric cancer cell line BGC823	40 *μ*M for 24 h	↑ ROS ➔ ASK1/MKK4/JNK pathway	--| cell proliferation ➔ apoptosis	[61]
	Osteosarcoma cell line MG63	80 *μ*M for 24 h	↑ ROS ➔ cytochrome *c*/caspase 3 pathway	--| cell proliferation ➔ apoptosis	[62]
	Primary cells of giant cell tumor	40 *μ*M for 24 h	--| NF-*κ*B pathway ➔ JNK pathway ↑ caspases 3 ↓ MMP9	--| cell proliferation ➔ apoptosis	[64]
	Monoblastic leukemia cell line THP1	50 *μ*M for 24 h	--| Akt/mTOR and Raf/MEK/ERK pathways	--| cell proliferation ➔ apoptosis	[65]
	Tongue cancer cell lines CAL27, HN21B, and HN96	100 *μ*M for 48 h	↓ MMP10	↓ cancer cells migration and invasion	[67]
	Breast cancer cell line MCF7	1–50 *μ*M for 24 h	↓ MMP9 --| PKC*α*, MAPK, and NF-*κ*B/AP1 pathway	↓ cancer cells migration and invasion	[68]
	Breast cancer cell line MDA-MB-231	10 *μ*M for 24–72 h	↓ MMP9 --| TGF*β*/Smad and TGF*β*/ERK pathways	↓ cancer cells migration and invasion	[69]
	Endometrial carcinoma cell line HEC1B	100 *μ*M for 24–72 h	↓ MMPs 2 and 9 --| ERK pathway	↓ cancer cells migration and invasion	[70]
	Colorectal carcinoma T-84 cells	50 *μ*M for 4 h	➔ PTPN1/cortactin axis	↓ cancer cells migration and invasion	[71]

CUR-PLGA	Prostate cancer cell lines C4-2, PC3, and DU145	30 *μ*M of CUR or equivalent CUR-PLGA for 48 h	↓ Mcl1, Bcl-xL, *β*-catenin, nuclear AR, STAT3, Akt, miR21 ↑ PARP cleavage, miR205	--| cell proliferation and ➔ apoptosis more efficient than native CUR	[83]

➔ induction; --| inhibition; ↑ increase; ↓ decrease. AP1: activator protein 1; AR: androgen receptor; ASK1: apoptosis signal-regulating kinase 1; Bax: Bcl2-associated X protein; Bcl2: B-cell lymphoma 2; Bcl-xL: B-cell lymphoma-extra large; ERK 1/2: extracellular signal-regulated kinases 1/2; JNK: c-Jun N-terminal kinase; MAPK: mitogen-activated protein kinase; Mcl1: myeloid cell leukemia 1; MKK4: MAPK kinase 4; MyD88: myeloid differentiation primary response protein; NF-*κ*B: nuclear factor *κ*B; PARP: polyadenosine diphosphate (ADP) ribose polymerase; PLGA: poly(d,l-lactic-co-glycolic acid); PTPN1: nonreceptor type 1 protein-tyrosine phosphatase; ROS: reactive oxygen species; STAT3: signal transducer and activator of transcription 3; TGF*β*: transforming growth factor *β*; TLR4: toll-like receptor 4; XIAP: X-linked inhibitor of apoptosis.
